# A 3D, Compartmental Tumor-Stromal Microenvironment Model of Patient-Derived Bone Metastasis

**DOI:** 10.3390/ijms24010160

**Published:** 2022-12-21

**Authors:** Mansoureh Mohseni Garakani, Megan E. Cooke, Michael H. Weber, Michael R. Wertheimer, Abdellah Ajji, Derek H. Rosenzweig

**Affiliations:** 1 Chemical Engineering Department, Polytechnique Montreal, Montreal, QC H3T1J4, Canada; 2 Institute of Biomedical Engineering, Polytechnique Montreal, Montreal, QC H3T1J4, Canada; 3 Department of Surgery, Division of Orthopaedic Surgery, McGill University, Montreal, QC H3G 1A4, Canada; 4 Injury, Repair and Recovery Program, Research Institute of McGill University Health Center (RI-MUHC), Montreal, QC H3G 1A4, Canada; 5 Department of Engineering Physics, Polytechnique Montreal, Montreal, QC H3T1J4, Canada

**Keywords:** 3D co-culture system, nanofibrous scaffolds, interface tissue models, drug screening, bone metastasis, personalized medicine

## Abstract

Bone is a frequent site of tumor metastasis. The bone–tumor microenvironment is heterogeneous and complex in nature. Such complexity is compounded by relations between metastatic and bone cells influencing their sensitivity/resistance to chemotherapeutics. Standard chemotherapeutics may not show efficacy for every patient, and new therapeutics are slow to emerge, owing to the limitations of existing 2D/3D models. We previously developed a 3D interface model for personalized therapeutic screening, consisting of an electrospun poly lactic acid mesh activated with plasma species and seeded with stromal cells. Tumor cells embedded in an alginate-gelatin hydrogel are overlaid to create a physiologic 3D interface. Here, we applied our 3D model as a migration assay tool to verify the migratory behavior of different patient-derived bone metastasized cells. We assessed the impact of two different chemotherapeutics, Doxorubicin and Cisplatin, on migration of patient cells and their immortalized cell line counterparts. We observed different migratory behaviors and cellular metabolic activities blocked with both Doxorubicin and Cisplatin treatment; however, higher efficiency or lower IC50 was observed with Doxorubicin. Gene expression analysis of MDA-MB231 that migrated through our 3D hybrid model verified epithelial–mesenchymal transition through increased expression of mesenchymal markers involved in the metastasis process. Our findings indicate that we can model tumor migration in vivo, in line with different cell characteristics and it may be a suitable drug screening tool for personalized medicine approaches in metastatic cancer treatment.

## 1. Introduction

Bone is a frequent site of tumor metastasis when primary tumors spread from their original site to the bone. Bone metastasis is common following primary breast (65–75%), prostate (65–75%), thyroid (60%), lung (30–40%), bladder (40%), and kidney (20–25%) cancer [1,2]; and among all the bones, spine is the most common site [3,4,5,6,7]. It was estimated by the American Cancer Society that among 750,000 Americans diagnosed with breast, lung, and prostate cancer in 2021, around 60% of them will then develop bone metastases, commonly occurring in the spine [8]. Bone metastases can be osteoblastic, osteolytic or a mixture of both, characterized by deposition of the new dense bone and destruction of the bone, respectively. Both are caused through factors secreted by cancer cells, affecting the normal homoeostasis of bone formation and resorption, resulting in changes to the structure and function of the bone. Excessive bone formation or resorption leads to vertebral instability, fractures, high blood calcium levels, and spinal cord compression [9]. Current treatments are surgery, along with radiation therapy and systemic chemotherapy, which is associated with severe side effects. Overwhelmingly, with rare exceptions, bone or spine metastasis cannot be cured and long-term survival of patients with metastatic cancer is low [10]. Thus, more studies are necessary to have better understanding of the interaction between tumor cell and bone microenvironments, alongside better in vitro culture models to determine the best course of treatment based on a patient-to-patient approach.

General approaches of “one-size-fits-all” like the above-mentioned therapeutic strategies have been traditionally used to treat patients with cancer [11]. However, no two patients’ cancers are the same and will have variable responses to different chemotherapeutics and doses, as tumors may have different underlying genetic sources, and express various proteins from one patient to another. Precision or personalized medicine is an emerging predictive, preventive, and more tailored approach that can be unique to an individual patient’s needs based on the genetic profile of their cancer cells, overcoming limitations of standardized treatment [11,12,13,14]. Current personalized medicine approaches still effectively use 2D cell culture-based models in drug pre-clinical screening which cannot reflect the complexity and heterogeneity of the tumor microenvironments [15]. Additionally, animal xenografts, as commonly used models in pharmaceutical research, are poor predictors of drug safety in humans, along with other limitations, including ethical concerns, high cost, and time-consuming processes, which cause delays in drug approval [16]. Thus, bridging in vitro cell culture and in vivo animal models by developing proper 3D culture technologies is essential. Advanced 3D culture models provide more physiologically relevant environments to assess tumor heterogeneity. Further, 3D bioprinting approaches for personalized treatment [17,18,19,20], human organoid/tumoroid models [21,22,23,24,25,26,27,28], and microfluidic modeling of the tumor microenvironment [29,30,31,32] are some examples of recent state-of-the-art tools adopted in anti-cancer drug discovery and personalized therapy. However, few such 3D cancer models enable one to separate stroma/tumor tissue compartments so as to better mimic the tumor microenvironment. For example, (i) a breast cancer model was fabricated by 3D bioprinting, in which a “tumor” in the center was surrounded by a stromal compartment [33]; (ii) co-extrusion produced a core–sell structure with immune cells in the core and tumor cells in the outer layer [34]; (iii) multi-channel microfluidic devices combined with hydrogels filled with different stromal and breast cancer cells in separate channels [35,36,37,38,39,40] allowed mimicking the stroma–tumor environments of breast cancer and to subsequently assess their response to different chemotherapy drugs. 

Numerous natural and synthetic biomaterials have been developed for 3D cell culture or co-culture models for studying cancer metastasis [15,41,42,43]; however, only few have separated compartments with different mechanical characteristics mimicking soft-hard tissues interfaces. To close this gap, new strategies need to be introduced based on electrospinning combined with hydrogel, or 3D bioprinting of gradient-based material or multi-materials [42,44]. Such advanced stroma–tumor 3D models may potentially replace animal-based studies for developing new therapeutics and improving treatment efficacy with higher success rates in pre-clinical trials. In previous work [45], we presented a 3D interface model of the tissue–tumor microenvironment based on the combination of an electric discharge plasma-treated nanofibrous PLA scaffold pre-seeded with stromal cells, and an alginate/gelatin-based hydrogel embedded with tumor cells. The purpose of using hydrogel integrated in this model was to represent a soft tissue interface for tumor interacting with a stiffer microenvironment such as bone/cartilage/fascia, etc. (which is the nanofibrous mat in our case). Thus, we made an interface 3D model by using hydrogel incorporated with tumor cells (representative of soft tissue) overlaid on a nanofibrous mat seeded with stromal cells (representative of stiff tissue) to replicate a soft/hard tissue interface. This innovative model enables quantifying migration and chemotherapeutic response of different tumor cell lines towards a stromal comportment. In this current study, we greatly expand that earlier work: (i) we use patient-derived spine-metastasis tumor cells embedded in hydrogel, like an organoid; (ii) an interface is established with human primary bone cells, osteoblasts, pre-seeded in plasma-treated 3D fibrous scaffolds, all together reproducing a real bone–tumor tissue model; (iii) subsequent tumor cell invasion/metastasis is assessed through the model; (iv) two different chemotherapy drugs are used to screen the bone–tumor interface model, accompanied by studying gene expression profile of tumor cells. This can become a customizable, flexible platform (a) for oncologists to monitor and predict how the disease will progress; (b) to screen novel therapeutics or new repurposing chemotherapy combinations to help specific cancer patients.

## 2. Results and Discussion

### 2.1. Invasion Behavior of Patient-Derived Spine-Metastasized Cells in the PP-3D-S Model

**Figure 1:**
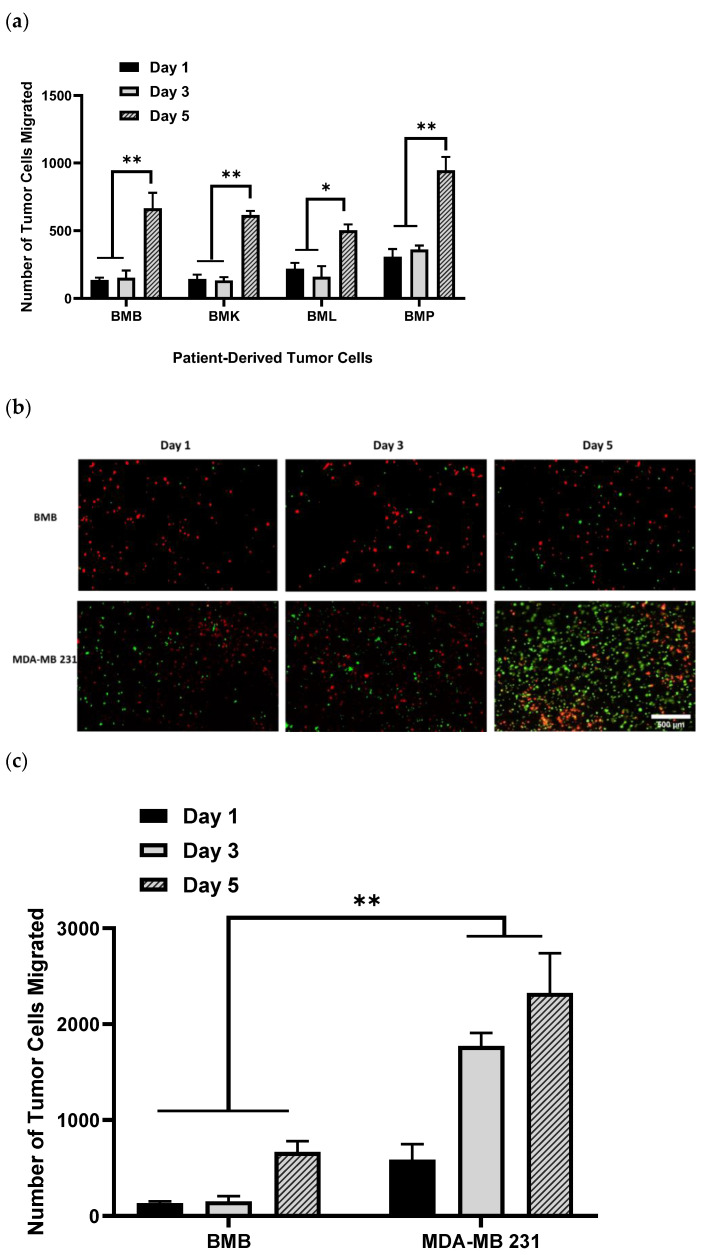
DiO-labeled patient-derived bone/spine-metastasized tumor cells secondary to Breast: Bone Met Breast (BMB), to kidney: Bone Met Kidney (BMK), to lung: Bone Met Lung (BML), and to prostate: Bone Met Prostate (BMP) cultured in hydrogel having migrated into osteoblast-seeded NH_3_ plasma-treated PLA scaffolds; (**a**): Histograms representing patient-derived bone-metastasized tumor cells numbers migrated to the bone interface, which is NH_3_-plasma treated PLA mats pre-seeded with DiI-labeled osteoblasts over the incubation time of 5 days; (**b**): Representative images of DiI-labeled red osteoblasts adhering to NH_3_ plasma-modified mats, along with DiO-labeled patient-derived metastasized cells and/or MDA-MB 231 (GFP) breast cancer cells having migrated after 5 days; (**c**): Comparison of patient-derived bone-metastasized breast cancer cells (BMB) with aggressive breast cancer cell line (MDA-MB 231) while migrating over 5 days*,* [scale bar: 500 µm, error bars: SE., * = *p* < 0.05, ** = *p* < 0.01, comparison indicated by lines].

Having previously examined our 3D model for migration and drug screening assay using different aggressive, less-aggressive, and non-cancerous breast epithelial cells lines [46], we sought to represent a real bone tissue–tumor interface model by reproducing it with patient-derived tumor cells, isolated from bony spinal metastases (BMX), including BMB, BMK, BML, and BMP. These BMX metastatic spine tumor cells all are aggressive, and we expected to see considerable migration and invasion over different time points. As illustrated in Figure 1a, we observed quite similar amounts of migration for all BMX samples, especially after 5 days, with no substantial difference between cells from different primary tumors individually at the certain time points. However, for each BMX sample, we found significant difference in the number of tumor cells migrated between day 1/3 and day 5. In addition, when they are compared with their equivalent cell lines, for example, BMB cells to their counterparts of MDA-MB 231 (micrographs of Figure 1b and histograms of Figure 1c), there is appreciable variance in the rate of migration and potential growth of BMB and MDA-MB 231 cell lines at the same time point; because patient-derived BMX samples are primary cells, they do not proliferate, migrate, and generally behave the same way that immortalized cell lines do. On the other hand, there might be a possibility of stromal cells migration up to the hydrogel as this reverse effect may have a potential confounding factor which must be addressed in future work. Moreover, since we quantified the number of cells (dots) at each time point, it might not be a representative of definite and pure migration. Indeed, migration analysis needs to be performed with appropriate software enabling live measurement of actual migration parameters, including displacement, speed, and track length, which would be considered as a potential caveat in our future experimental design.

In order to compare our PP-3D-S model with one of the most trending 3D cancer models, multi-channel microfluidic devices, used to screen chemotherapy drugs for cancer patient samples, both are able to be designed based on co-culture systems. Combined with hydrogels, both models are filled with different stromal and cancer cells while separating tumor–stromal tissue compartments [35,36,37,38,39,40]; it thus allows better mimicking of the tumor microenvironment and better assessment of the cellular response to different chemotherapeutics. The advantage of microfluidics is that those 3D models have more precise control over the microenvironment by resembling diffusion/gradients/perfusion and better simulating of physiological processes and vascularized tissues. In addition, the number of required samples can be reduced, and this technique needs 10 to 1000 times less sample volume than the other methods, thus testing drug compounds on live patient cells can be performed rapidly due to the small cell numbers [47,48]. However, microfluidic devices are difficult to scale-up and be adapted to high throughput screening in terms of the fabrication of chips. Moreover, PDMS chips are hydrophobic and require surface modification for subsequent cell seeding, and the 3D tumor formed in the microfluidics remains a challenge to be removed from the chips for further analysis, particularly immunofluorescence imaging [49]. When compared to PP-3D-S, as mentioned before, our model can be customized to present a soft–hard tissue interface matched with the target tissues by using a broader range of substrate stiffness, which is not applicable by microfluidic devices. Our model is fast and easy to use in terms of manipulation and it helps improve reproducibility since it avoids the characteristic of batch-to-batch variation observed by natural hydrogels like Matrigel; it is also able to be tailored to a high-throughput format because our 3D system is compatible with liquid-handling equipment or the 3D bioprinting technique. However, to accurately represent a realistic and more physiologically relevant tissue–tumor model, it still needs to be modified in several aspects, particularly incorporating vascular endothelial cells to the stromal compartment of the 3D model which is considered an important part of mimicking the extravasation process. It may also be beneficial to use diverse populations of matched patient-derived stromal cells while seeding patient-derived tumor cells in the tumor compartment to replicate the tumor microenvironment and subsequent chemotherapy drug screening tests more accurately. Moreover, in order to better mimic the dynamic physiological conditions of the natural microenvironment, our static model can be integrated with a bioreactor with fluid flow-induced shear stress or microfluidics to overcome this limitation.

### 2.2. Screening of Patient-Derived Bone-Metastasized Cells with Doxorubicin

**Figure 2:**
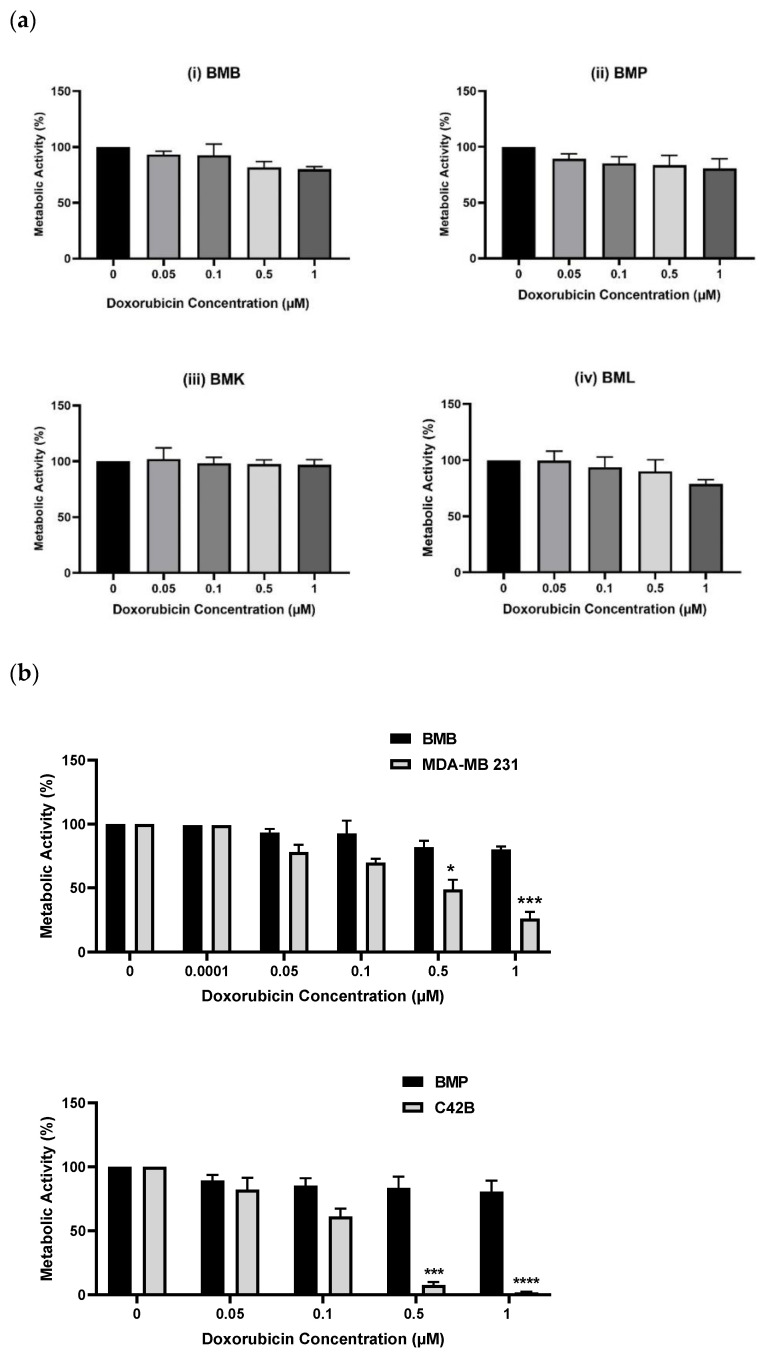
Metabolic activities of (**a**): Different patient-derived bone/spine-metastasized tumor cells secondary to Breast: BMB (i), to prostate: BMP (ii), to kidney: BMK (iii), and to lung: BML (iv) screened with Doxorubicin (0, 0.05, 0.1, 0.5, and 1 µM) after 5 days, (**b**): Two pairs of patient-derived cells and their equivalent immortalized cell lines including BMB and MDA-MB 231, and MBP and C42B after 5 days of culture; [error bars: SE., * = *p* < 0.05, *** = *p* < 0.001, **** = *p* < 0.0001, bars with “*” are compared with control (Dox 0) and/or with their equivalent values of BMX samples].

The end purpose of developing this bone or spine metastasis 3D model with primary patient-derived cells is their application in drug screening tests for personalized medicine, to screen novel chemotherapeutics or repurposing chemotherapy combinations, to help patients suffering from cancer. We conducted screening tests of the above-mentioned patient-derived BMX cells with Doxorubicin (Dox) and compared the results with their equivalent cell lines at the similar range of drug concentration. From Figure 2a, we observed that BMX cells were protected from Dox-induced apoptosis, meaning that they were resistant to Dox at the same doses of 0, 0.05, 0.1, 0.5, and 1 µM, which had been used earlier for the corresponding cell lines [46]. Further, we compared the differences between patient-derived cells and their equivalent cell lines including BMB and MDA-MB 231 breast cancer cells line and/or BMP and C42B prostate cancer cells line by assessing drug sensitivity, as shown in Figure 2b. The results indicated remarkable resistance of patient-derived bone metastasized tumor cells to Doxorubicin compared with consistent drug sensitivity of their counterpart cell lines at the same drug concentrations. This is meaningful data, achieved by applying our PP-3D-S model, showing substantial differences between the response to chemotherapeutics of immortalized cell lines versus patient-derived cells.

### 2.3. Treatment of Bone-Metastasized Secondary to Breast Tumor Cells with Different Chemotherapy Drugs

Metastasis and drug resistance are among the major issues to the treatment of advanced cancer, and the leading causes of death in cancer patients. Anti-cancer drugs discovery and development is still the main area of cancer research. It is clear that molecular and phenotypical changes induced by different chemotherapy drugs that have effect on intra-cellular signaling pathways, cell division, and -proliferation are specific for each particular cell type [50]. Doxorubicin and Cisplatin (Cis) are both considered to be effective, and they are commonly used chemotherapy drugs, among others, to treat cancer. With the aim of investigating drug resistance/sensitivity of the targeted tumor cells, we carried out a series of experiments on either patient’s breast cancer cells metastasized to bone (BMB), or an equivalent cell line, MDA-MB 231, screened with both Dox and Cis at different doses over the course of 5 days. It seems that a 5-day period for the cell culture is a very short observation interval for such complex processes as invasion, migration, and metastasis. However, the reason that 5 days of culture time was chosen for both migration and drug screening tests is due to commercial purposes. From an industrial point of view, time is very important for high-throughput screening tests, and thus, we preferred to focus on screening cells with chemotherapy drugs within a short time (5 days) to assess the effectiveness of the drugs on blocking tumor cells (this research has been already filed as a patent).

**Figure 3:**
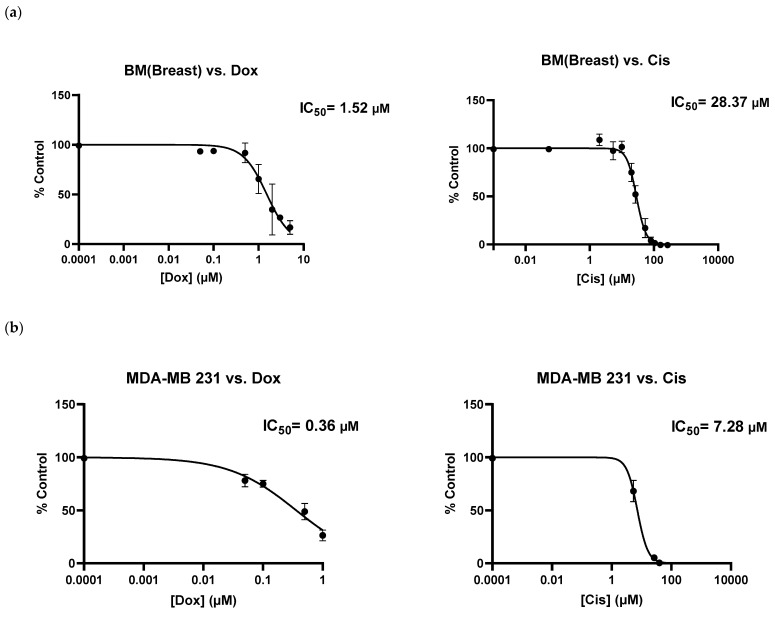
Metabolic activities of (**a**) patient-derived bone-metastasized tumor cells secondary to Breast: BMB and (**b**) MDA-MB 231 breast cancer cell lines screened with Doxorubicin (0–5 µM) and Cisplatin (0–200 µM) after 5 days of culture; [error bars: SE.].

As shown above (Figure 2a), BMB cells were resistant to Dox concentrations between 0.05 to 1 µM. However, by increasing its concentration from 1 to 5 µM (Figure 3a), we observed a marked reduction in metabolic activity of BMB cells, with the relative IC50 value around 1.52 µM. It is interesting to compare this with the IC50 value of MDA-MB 231, 0.36 µM, which clearly shows an important difference in drug sensitivity between BMB patient-derived cells and their equivalent MDA-MB 231 breast cancer cell line. When compared to Cisplatin and the other chemotherapeutic drug used in this study, there was also a considerable difference in chemosensitivities between the two drugs after screening both MDA-MB-231 and BMB cells with Dox and Cis: Comparing the two curves shown in Figure 3a, it is clear that BMB cells are more sensitive to Dox (IC50 = 1.52 µM) than to Cis (IC50 = 28.37 µM) at the same drug concentrations, while Figure 3b reveals a similar trend for the immortalized MDA-MB 231 breast cancer cell line, namely IC50 values of 0.36 and 7.28 µM for Dox and Cis, respectively. We believe that there might be a crucial connection between molecular states before drug treatment and cellular phenotypes induced by different chemotherapeutics after treatment, one that needs to be transcriptionally investigated in our future studies [51,52,53,54,55,56,57,58]. The results above clearly underline that our current 3D culture model is appropriate for screening not only cell lines, but also different patient-derived metastasis cells with a variety of existing or newly developed chemotherapy drugs for specific patients.

We also compared our 3D model with 2D culture while screening different more- or less-aggressive immortalized breast cancer cell lines and non-cancerous breast epithelial cells with Doxorubicin through calculating the values of IC50 (data provided in Appendix A, Figure A6). IC50 values obtained by the 2D model were less than those of our 3D model, as expected. Given the meaningful difference in the IC50 value achieved, we demonstrated that our 3D system is advantageous over 2D, indicating that cells show more physiological behavior in the 3D environment. We could not provide data of patient-derived metastasized cells screened with Doxorubicin in the 2D model since we did not have enough cells but have done it in past work [59].

### 2.4. Gene Expression Profile of Breast Cancer Cell Lines in the PP-3D-S Model

**Figure 4:**
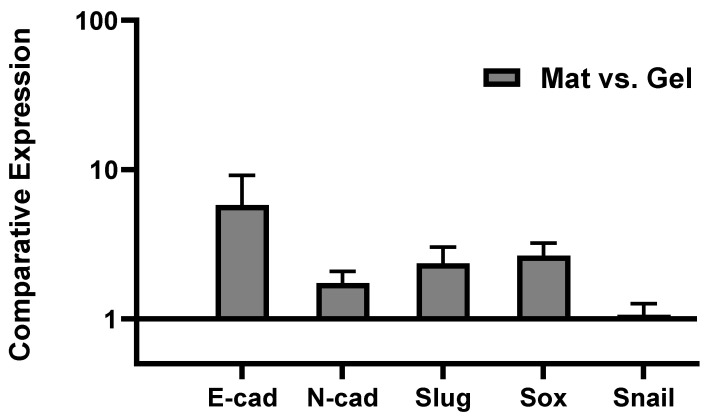
Gene expression profile of MDA-MB 231 aggressive breast cancer cells cultured in PP-3D-S model alone (without stromal osteoblasts/fibroblasts) for 5 days: expression of E-cadherin, N-cadherin, Slug, Snail, and Sox markers by tumor cells that had migrated from hydrogel onto the mat (stromal cells) while undergoing EMT mechanism versus tumor cells remained in hydrogel quantified by real-time qPCR assay; [error bars: SE.].

Epithelial-to-mesenchymal transition (EMT) and the reverse process (MET) have been widely discussed in the literature as key steps in cancer metastasis: They are biological processes by which cancer cells transit between epithelial and mesenchymal states [59]. Indeed, immotile epithelial cells undergo multiple biochemical changes and specific morphological alterations in which they lose their tight cell–cell adhesion, switch to mesenchymal phenotypes, and eventually acquire enhanced motility, migratory properties, and invasiveness. Following travel (metastasis) to a new location, multiple steps occur for the metastatic cancer cells to recover to their epithelial characteristics, termed MET, and form a secondary tumor [60,61,62]. Several different biomarkers/signaling pathways and molecular processes involved in initiation of EMT have been proposed in the literature, including expression of cell-surface proteins/markers of EMT: E-cadherin, N-cadherin [63,64,65]; cytoskeletal markers of EMT: Vimentin [66]; extracellular proteins: Fibronectin [67]; and activation of transcription factors: Snail, Slug, Zeb, Twist, and Sox families [68,69,70,71]. These markers all are upregulated during cancer metastasis, known as mesenchymal markers, except E-cadherin (cell-cell attachment receptor) which is decreased during EMT as epithelial markers [61,62]. Since we observed significant MDA-MB 231 migration after 5 days (quantitatively proved in Figure 2c), we investigated the possible role of EMT mechanisms through monitoring the expression of E-cadherin, N-cadherin, Twist1, Slug, Snail1, and Sox2 by tumor cells by means of real-time qPCR. As mentioned in the methods, the 3D hybrid model was seeded only with MDA-MB 231 tumor cells, and thus the result from qPCR tests is just presenting the genes expressed by tumor cells in the course of migrating from the hydrogel to the mat while undergoing the EMT mechanism versus those tumor cells that remained in the hydrogel. The reason that the model was cultured only with tumor cells is that by running the model with both stromal cells and tumor cells, it would not be possible to distinguish stromal and tumor cells, including those migrated tumor cells after 5 days of incubation. An option would be to use cell sorting. Both stromal (RFP) and tumor (GFP) cells could be detached from the mats by using trypsin and then sorted by the FACS machine. Future work will attempt to optimize this approach to use co-culture and distinguish effects of multiple cell types. To clarify again, in this paper, the PP-3D-S setup was adjusted based on using only tumor cells for gene expression analysis. Figure 4 shows epithelial and mesenchymal markers expressed by MDA-MB 231 cells that migrated onto the mats, compared with the cells that remained in the hydrogel (data normalized to the gel). Clearly, an appreciable proportion of tumor cells that had migrated into the mats manifested increased expression of mesenchymal markers N-cadherin, Slug, and Sox2 compared with the values in the gel, the expected upregulation of those markers during EMT. However, we found that expression of E-cadherin, the epithelial marker, also showed great increase. This indicates that cells which readily migrated to the mats not only lost their E-cadherin receptors while leaving the gel and acquiring the mesenchymal phenotype while undergoing EMT, but that they also might be able to revert to their original phenotype. Termed MET, this occurs in the secondary environment, the mat in this case, after cell growth and proliferation on the mat over 5 days of culture [72]. Nevertheless, some limitations remain as we only investigated one tumor cell line and one time point. Thus, future work will have to explore a time course with several tumor cell types to fully understand the impact of EMT on our 3D model. Taken together, these findings highlight that this PP-3D-S in vitro 3D culture model indeed can realistically represent cancer cell migration/invasion processes; here, EMT was manifested by overexpression of mesenchymal markers. Nevertheless, besides numerous published reports in the literature proving that EMT is crucial in metastatic cancer, alternative mechanisms have also been proposed for spreading and metastases. These include collective or cluster-based migration, mesenchymal invasion, and amoeboid invasion (crawling-like cell movement) [73]. 

## 3. Materials and Methods

### 3.1. Fabrication of Plasma-Treated Electrospun Nanofibrous Scaffold

#### 3.1.1. Electrospinning of 3D Electrospun Mat

We fabricated poly (lactic acid), PLA (NatureWorks 4032D, density = 1.24 g/cc) randomly oriented nanofibrous scaffolds with fiber diameters between about 600–800 nm. Details in the fabrication of all three different fiber diameters, “Small”: 200–300 nm; “Medium”: 600–800 nm; and “Large”: 1–2 µm made of various polymers have been previously published [45,46]. Briefly, 16 wt.% polymer solution containing PLA pellets dissolved in 2,2,2 trifluoroethanol, TFE, (M = 100.04 g/mol, Merck, Billerica, MA, USA) solvent was electrospun; to fabricate mats of 250 μm nominal thickness, 10 mL of polymer solution was fed at a flow rate of 1.6 mL/h using a syringe pump, all placed inside a chamber with controlled temperature (21–24 °C), and relative humidity of 45–50%. The distance between the grounded needle tip (21G) and a rotating mandrel (25 rpm) was set at 15 cm, and a stable DC high-voltage (HV) power supply provided a voltage of 20 kV between them. The rotating metal drum mandrel, pre-wrapped with an aluminum foil, was used to collect nanofiber filaments, the finished electrospun mats on Al foil then being maintained in ambient air for 3 days to evaporate residual solvent, then gently detached, cut into smaller pieces, and stored in a desiccator for subsequent use. 

#### 3.1.2. Surface Treatment of 3D Scaffolds by Plasma Functionalization 

The fiber surfaces throughout the highly porous (ca. 90%) open volume of the fabricated 3D electrospun mat were exposed to low-pressure radiofrequency (rf) glow discharge plasma in NH_3_ gas; based on data from earlier work [45,46] PLA mats so-treated were optimized for tumor cell migration/adhesion. Since much detail has already been described [74,75,76], we explain here only essential aspects. As stated above, the mats were functionalized using ammonia gas (Air Liquide Canada Ltd., Montreal, QC, Canada) in a low-pressure (600 millitorr or 80 Pa) capacitively coupled radiofrequency (r.f., 13.56 MHz) glow discharge plasma reactor (cylindrical aluminum/steel chamber) with a gas flow rate of 15 standard cubic centimeters per minute (sccm) and plasma exposure time of 1 min under mild plasma conditions (power: 15 W and Voltage: −40 V). The treated mats were then stored in sealed sterilized Petri-dishes before subsequent experiments and analysis. 

**Figure 5:**
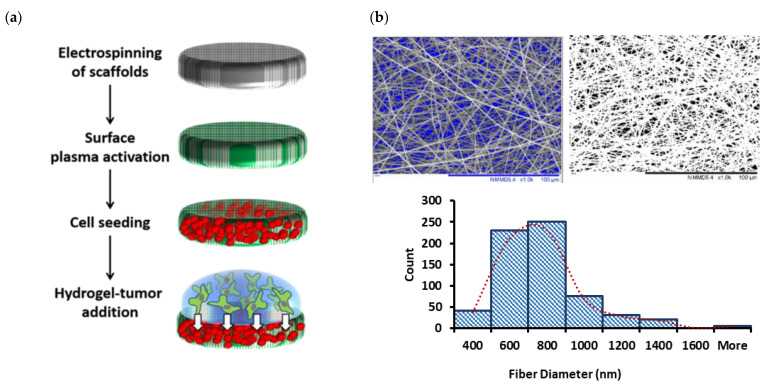
(**a**): Schematic diagram of the main steps of the current “PP-3D-S” 3D interface model; (**b**): SEM micrographs of medium-sized electrospun PLA mats (fiber diameter sizes: 730 ± 75 nm; Average pore size: 2.1 ± 0.2 µm; overall porosity: 91.0 ± 1.6%) along with fiber diameter distribution curve; scale bar: 100 µm (adapted from [45]).

More details of processing parameters and methods for measuring mats thickness, fiber diameters, pore sizes, and overall porosity of nanofibrous mats, along with explanation on different plasma techniques used for surface treatment and surface physio-chemical characterization results for non-treated and variously plasma-treated 3D scaffolds were presented earlier [45]. Figure 5 will familiarize the reader with (a) the overall process scheme, the “PP-3D-S” model; (b) the 3D mat type exclusively used in this present article. 

### 3.2. Biological Experiments 

#### 3.2.1. Cell Culture and Seeding in Hybrid 3D Scaffolds

Referring to (a) of Figure 1, the “stromal” (fibrous mat) component of the “PP-3D-S” model [45,46] (hereafter part “A”) was prepared as follows: primary human osteoblasts were isolated from the vertebral bodies of healthy organ donors (REB Extracellular Matrix Protocol # 2020-5647, approved by the institutional review board of McGill University) labeled with DiI (red) membrane labeling dye (Molecular Probes, Invitrogen, according to manufacturer’s instructions) and IMR-90 mCherry-labeled fibroblasts (red florescent), provided by the laboratory of professor M. Park at McGill University, were seeded onto the sterile electrospun scaffolds 24 h after plasma treatment. For the “tumor” component (hereafter, part “B” of the 3D model), triple negative epithelial breast cancer cell line MDA-MB231 (Green-fluorescent protein, GFP), donated by Park lab, as well as C42B prostate cancer cell line, and a series of the following patient-derived metastatic tumor cells all from consented donors at the MUHC (RI-MUHC, REB Extracellular Matrix Protocol # 2020-5647) were labeled with DiO (green) membrane dye (Molecular Probes, Invitrogen): patient-derived cells of bone metastasized secondary to breast (Bone Met Breast, BMB), to prostate (Bone Met Prostate, BMP), to lung (Bone Met Lung, BML), and to kidney (Bone Met Kidney, BMK). They were then embedded into a mixture of hydrogel (A1G7) based on 1% wt. Sodium Alginate (Protanal LF10/60, Pharm grade, FMC) and 7% wt. Gelatin (G9391 from bovine skin, type B, Sigma, Saint Louis, MO, USA) to mimic a bone-metastasized tumor microenvironment. Cells were cultured in high-glucose Dulbecco’s modified Eagle medium (DMEM), supplemented with 10% fetal bovine serum (FBS) and 1% penicillin/streptomycin (PS) (all from Gibco, Thermofisher, Grand Island, NY, USA), at 37 °C in a humidified incubator with an atmosphere of 5% CO_2_. Plasma-treated electrospun scaffolds (Part A) were cut into disks with a 9 mm biopsy punch, disinfected with RPMI media containing 1% antibiotic, then placed into the wells of non-adherent 48-well polystyrene culture plates (SARSTEDT AG & Co., Nümbrecht, Germany). Then, 20,000 RFP-Fibroblasts or Dil-labeled osteoblasts were added to each well and incubated for 30 min at 37 °C and 5% CO_2_, following which the liquid was aspirated from each well and scaffolds were rinsed with fresh media to remove non-adhering cells. Finally, (Part B) 50,000 cells/well (either breast cancer cell line or patient-derived bone-metastasized cells) in a volume of 100 µL A1G7 hydrogel, were applied on top of the pre-seeded nanofibrous mats (A), followed by addition of 200 µL of 100 mM CaCl_2_, crosslink agent (Calcium chloride dihydrate, C7902, Sigma, Saint Louis, MO, USA) for ionic crosslinking. After 10 min CaCl_2_ was aspirated, scaffolds were washed twice with fresh media to remove residual crosslinking agent, and 500 μL of complete media (RPMI with 10% FBS and 1% PS) was added per well. The scaffolds were incubated for 1, 3, and 5 days (depending upon the specific experimental design), and the culture medium was changed every three days.

**Figure A1:**
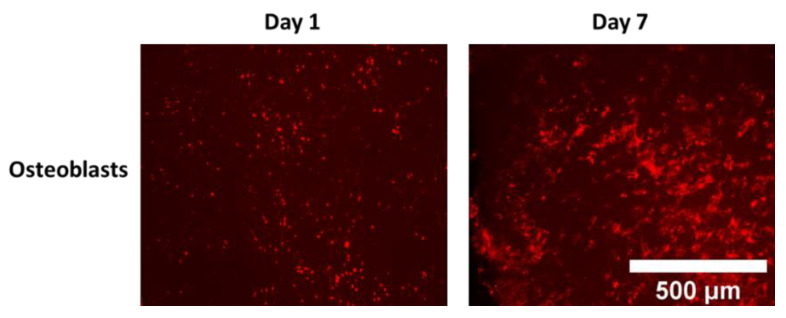
Representative images of Cell distribution/proliferation over 7 days of culture in NH_3_ plasma-treated PLA electrospun mats (top surface imaged by florescent microscopy).

**Figure A2:**
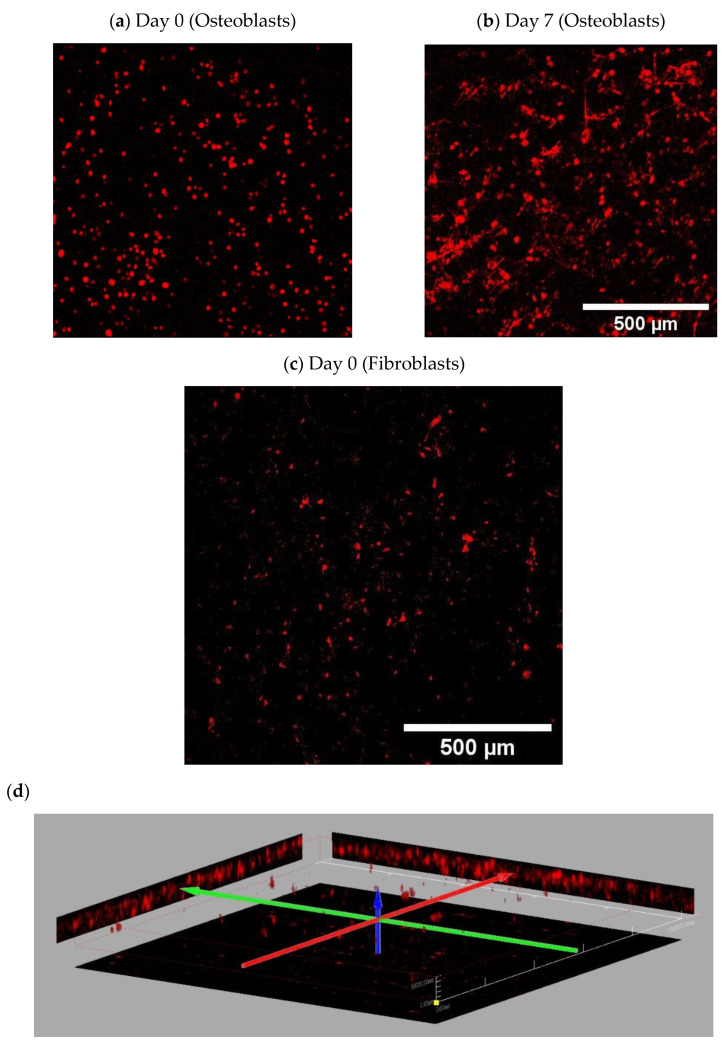
Representative images (3D stacked by confocal) of stromal cells seeded on NH_3_ plasma-treated PLA mats; Osteoblasts (DiI-labeled cells): (**a**) at day 0 (imaged after 30 min incubation with cells, penetration depth: max 60 μm) (**b**) after 7 days (penetration depth: max 120 μm); Fibroblasts (RFP): (**c**) at day 0 (imaged 30 min after incubation with cells, penetration depth: max 60 μm); (**d**) 3D projection of fibroblasts in depth of scaffolds.

**Figure A3:**
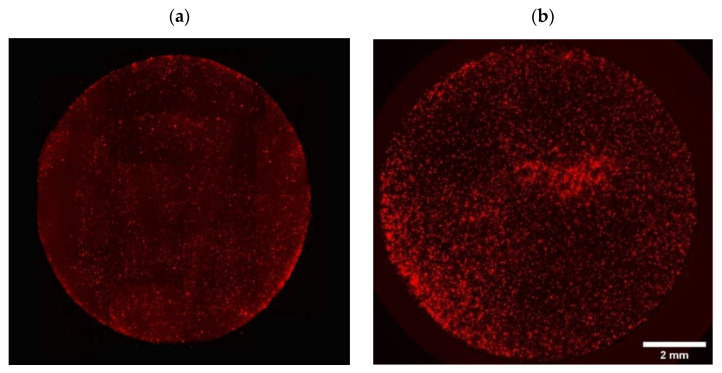
Representative fully covered images of initial cell adhesion on the NH_3_ plasma-treated PLA mats seeded with (**a**) fibroblasts (RFP) and (**b**) Osteoblasts (DiI-labeled cells).

**Figure A4:**
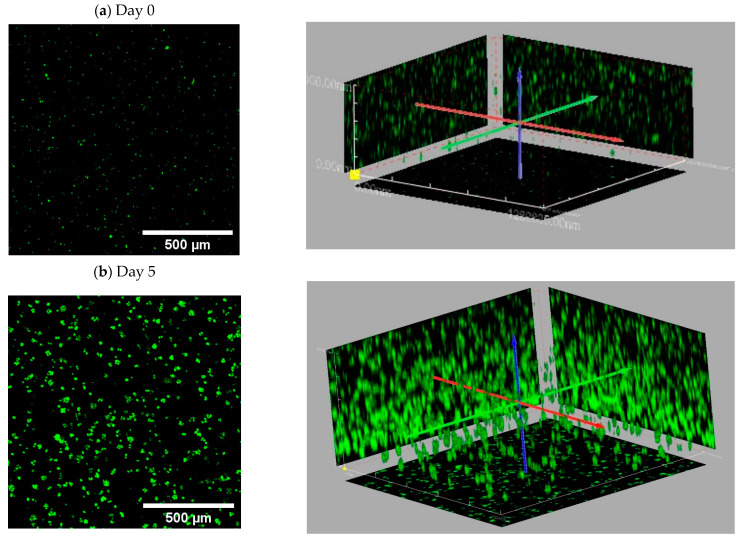
Representative 3D stacked images of MDA-MB 231 (GFP) cell distribution and proliferation in A1G7 hydrogel overlaid on mats over 5 days of culture: (**a**) day 0, after the time of gelling; (**b**) at day 5.

**Figure A5:**
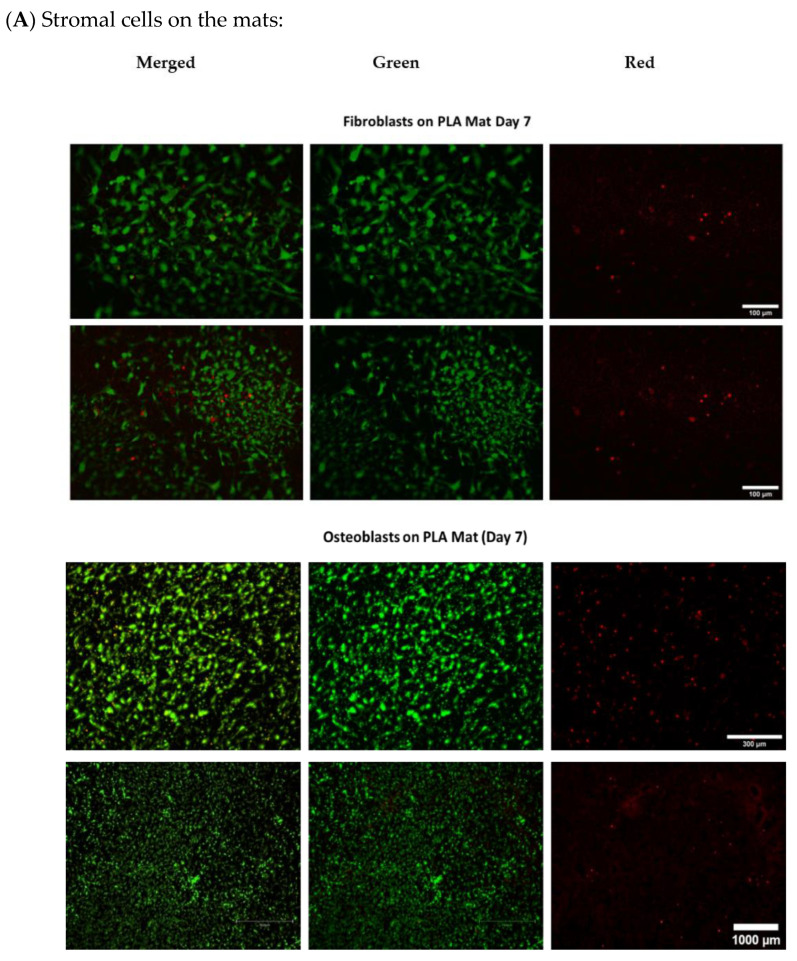

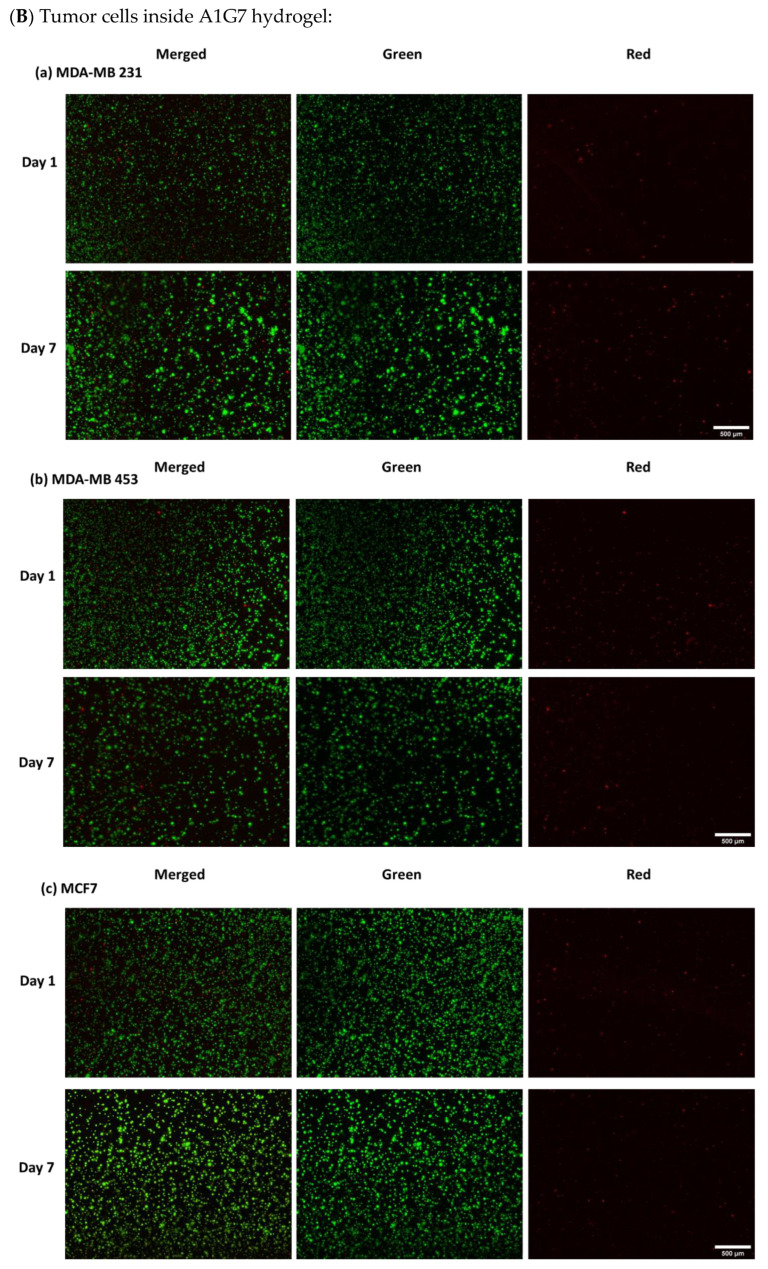

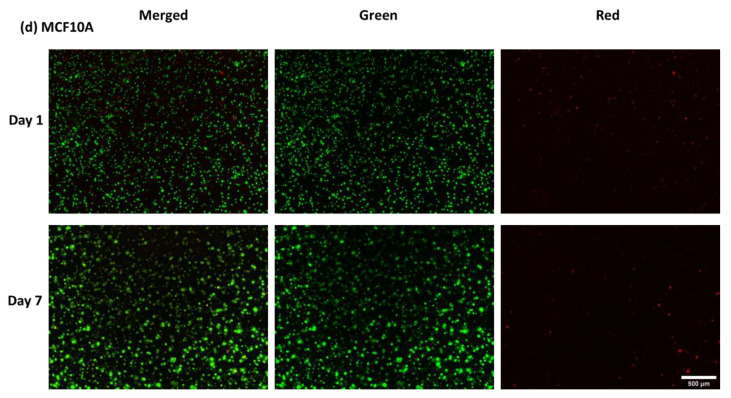
Live/Dead assay (**A**) Osteoblasts and fibroblasts cultured on NH_3_ plasma-treated PLA mats (**B**) different cancer cell lines in A1G7 hydrogel over 7 days.

**Figure A6:**
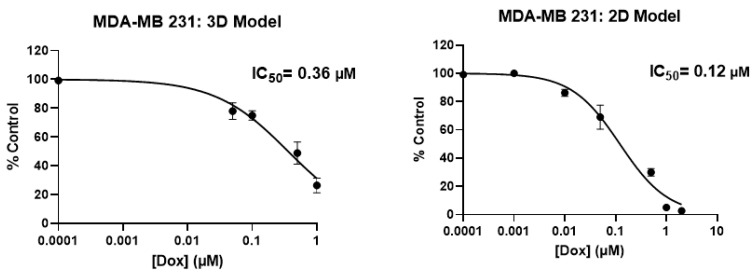

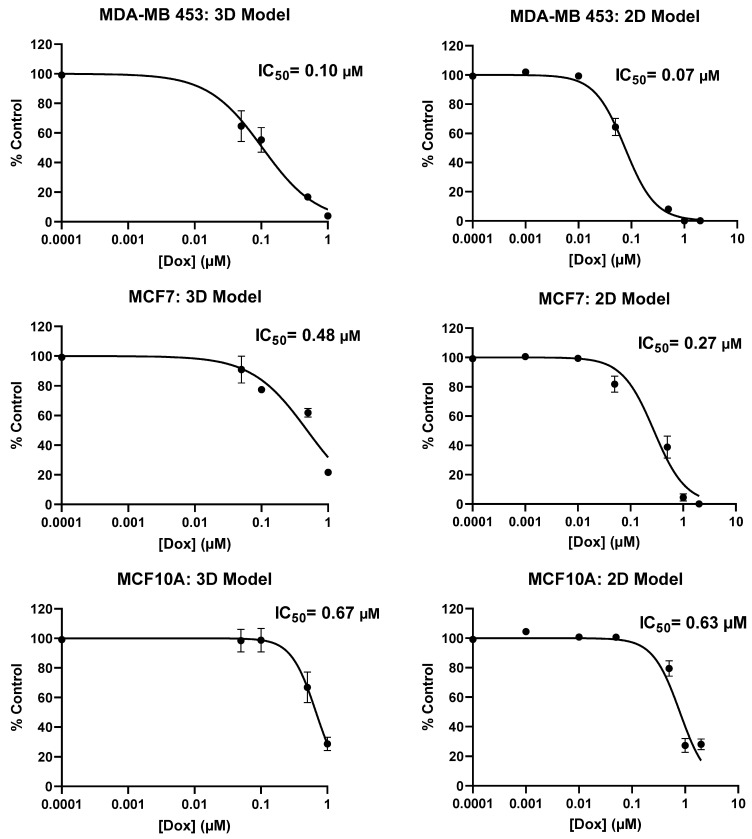
Comparison of metabolic activity (IC50 Value) of aggressive and less-aggressive breast cancer cells and non-cancerous epithelial breast cells in 2D and PP-3D-S model.

A series of cell viability tests (Live/Dead Assay) and cells distribution both on the mats (seeded with stromal cells) and inside the hydrogel (embedded with tumor cells) after 7 days of culture have been presented in Appendix A, Figure A1, Figure A2, Figure A3, Figure A4 and Figure A5.

#### 3.2.2. Observation and Quantification of Tumor Cell Migration

Migration from (B) and invasion of tumor cells into (A) was assessed as a function of incubation time. At different time points (over the course of 1, 3, and 5 days), cells on the treated mats were fixed with 4% Paraformaldehyde (PFA) after carefully removing the hydrogel off the surface. The samples were then placed on microscope slides covered with a drop of mounting medium (Sigma, Fluoroshield with DAPI), along with a protective glass cover slip to avoid dehydration. The top surface of the mats was monitored using florescent scanning microscopy (EVOS M5000 (2X)). The number of GFP/DiO labeled tumor cells present on top of the scaffolds were counted (20 spots/sample of three replicated experiments) using ImageJ (bundled with 64-bit Java 8, National Institute of Health, Bethesda, MD, USA). Each experiment was done in triplicate, with quantification from 20 spots to fully cover the surface of each sample. 

#### 3.2.3. Drug Screening Experiment

Doxorubicin (Dox) and Cisplatin (Cis), well-known chemotherapy drugs, were used in screening experiments designed to evaluate their effect on cellular activities of patient-derived metastasized cells seeded in our 3D cell culture model. NH_3_ plasma-treated PLA mats (used 24 h after plasma treatment) pre-cultured with stromal and tumor cells in 48-well plates were treated either with sterile PBS as control (placebo), or with Dox (0.05, 0.1, 0.5, 1, 2, and 5 μM) and/or Cis (1, 5, 50, 100, 150, 200 μM) in low-serum RPMI media (1% FBS, 1% PS) and incubated for 5 days. The experiment was performed with technical replicate of six and the media loaded with the drugs was replaced after 3 days. Cellular metabolic activity was analyzed by Alamar Blue assay after 5 days of treatment with Dox/Cis, and IC50 values (50% inhibitory concentration) were calculated by constructing a dose-response curve and using non-linear regression (Equation: log(inhibitor) vs. normalized response) in GraphPad prism. Finally, the numbers of migrated cells from the gel (Part B) to the surface of mats were measured by florescent microscopy and quantified using ImageJ (Java 8).

#### 3.2.4. Metabolic Activity Measurement

Cellular metabolic activity was assessed using a commercial Alamar Blue^®^ kit (Thermofisher), whereby resazurin dye (blue) is reduced to resorufin (pink). Briefly, stromal and tumor cells cultured in the 3D model and treated with Dox or Cis (for 5 days) were incubated with Alamar Blue dye added to media (RPMI, 1% FBS, 1% PS) at dilution of 1:10 at 37 °C, 5% CO_2_ for 4–8 h, depending on the cell type. After loading a volume of 100 µL from each well into 96-well plates (Corning, black half-area), fluorescence of Alamar Blue at Excitation/Emission wavelengths 540/585 nm were analyzed using a Tecan Infinite M200 pro microplate reader (Tecan Trading, AG, Männedorf, Switzerland). The experiments were performed in triplicate for each cell type to assure reproducibility.

#### 3.2.5. Gene Expression Analysis: Real-Time qPCR

For qPCR analysis, the model was run only with MDA-MB 231(GFP) tumor cells embedded in A1G7 hydrogel while overlaying on the empty mats (without stromal cells). After 5 days of incubation, RNA from tumor cells remaining in the hydrogel and from those that had migrated to the mat’s surface were separately isolated using Trizol reagent (Ambion, Life technologies). The manufacturer’s protocol was followed: Trizol was added, and the scaffold disrupted through mechanical homogenization, before RNA was separated with chloroform, precipitated by isopropanol, washed with 75% ethanol (diluted in ultra-purified water), and finally dissolved in RNase-free water. RNA concentration and quality were determined by spectrophotometry (Tecan). Then, 400 ng of total RNA was reverse transcribed into cDNA using a qscript cDNA synthesis kit, according to the manufacturer’s instructions (Quanta bio). Synthesized cDNA was then amplified, and gene expression measured using real-time-quantitative PCR (Applied Biosystems). Housekeeping genes and genes of interest were purchased from Invitrogen and primer sequences are listed in Table 1. To prepare a total volume of 20 μL, 10 μL SYBR Green Master Mix qRT-PCR kit (Thermo Fisher, Austin, TX, USA), 1 μL of cDNA, 0.5 μL of forward primer, 0.5 μL of reverse primer, and 8 μL of RNase free water were mixed. Then, qPCR was performed as follows: pre-denaturation for 10 min at 95 °C, denaturation for 15 s at 95 °C, annealing for 1 min at 60 °C, elongation/extension for 30 s at 72 °C, 40 cycles. Quantitative data were analyzed by average of duplicate Ct values, normalized to β-actin levels as an internal control, and target gene expression was calculated using the 2^^(–ΔΔct)^ method. The results represent three independent experiments, and the final values compare EMT markers expressed by MDA-MB 231 tumor cells that had moved out of hydrogel and migrated onto the mats relative to their expression by tumor cells remained in the hydrogel.

### 3.3. Statistical Analysis

All quantitative results reported here were obtained from at least three independent experiments to evaluate reproducibility, and data were expressed as the mean values ± SD. Statistical analysis was carried out using one-way ANOVA with Tukey’s post hoc analysis for parametric data; in the case of having non-parametric data, Kruskal–Wallis test was done, followed by Mann–Whitney post hoc analysis to compare two independent groups of interest. *p* values of less than 0.05 (*p* < 0.05) were considered significant for all tests. All statistical analysis was performed using Prism v 9.0 (GraphPad).

## 4. Conclusions

In this study, we adopted our previously developed PP-3D-S co-culture tumor microenvironment model [45,46] to investigate migration, metabolic activity, and drug sensitivity of patient-derived spine/bone metastases cells. Our 3D model could truly represent a physiological tumor microenvironment based on a stroma–tumor interface, which can be adaptable to different tissue porosity and mechanics for studying a broad range of cancer types. In future research, we propose that aside from fibroblasts or osteoblasts, two more stromal cell types, endothelial and immune cells, can be also seeded in the stromal compartment of the 3D system (electrospun nanofibrous mesh). Their co-culture with tumor cells in the second compartment (hydrogel) may more accurately replicate the tumor microenvironment and metastasis process with having a diverse population of stromal cells around tumor cells. It is known that endothelial cells and surrounding blood vessels/vascularization are critical parts of the event and play key roles in metastatic cascade when cancer cells detach from the primary tumor, intravasate into blood vessels and lymphatic systems. Thus, when endothelial cells are involved in our 3D model, it might better recapitulate essential aspects of metastasis cascade including intravasation and extravasation [77,78,79]. In the case of using patient-derived tumor cells in hydrogel, including matched patient derived stromal cells on the mesh may be of benefit here as well. The multi-well plate format on which the technology was built, combined with liquid-handling compatible hydrogel, make this novel platform a scalable and potentially high-throughput in-vitro model with several benefits over available 3D cell culture matrices such as Matrigel^®^ with its high cost and batch-to-batch variability. Ultimately, our scalable 3D in-vitro model is ideal for drug discovery and compound testing workflows and can be valuable for use in both academic and industrial labs.

Our model presented here, enables observing migration of patient-derived spine-metastasized cells secondary to breast, prostate, lung, and kidney cancers toward a stromal interface (Osteoblasts), although not to the same extent as immortalized cell lines, likely due to their lower metabolic activity. When challenged with the commonly used chemotherapeutics, Doxorubicin (Dox) and Cisplatin (Cis), patient-derived cells were found to be significantly more resistant to these drugs compared to immortalized cell lines. Patient-derived bone-metastasized breast cancer cells and breast cancer cell lines were both more resistant to Cis than to Dox, displaying higher IC50 values. Furthermore, we assessed common EMT markers (N-Cadherin, Snail, Slug, Sox2, and E-Cadherin) comparing expression levels in cells which migrated to the stomal compartment versus those remining behind in the gel after 5 days culture. Compared to the non-migrated cells, the migrated cells displayed higher expression N-cad, Slug, and Sox2, indicating the EMT status of the migrated MDA-MB-231 cells. However, there was also higher E-cad detection (epithelial marker), indicating that caution should be exercised since only one tumor cell line and one time point was probed. Taken all together, we have demonstrated that this 3D co-culture model, PP-3D-S, is sensitive to different immortalized and primary cell types, and that it provides a powerful environment in which patient-derived cells can be screened with different chemotherapeutics. It has strong potential as a tool to assess effectiveness of new drugs or drug combinations, to move towards the goal of personalized cancer treatment. 

## Figures and Tables

**Table 1:** Detailed information of primer sequences used for qRT-PCR analyses.

Full Name	Forward Primer 5′ > 3′	Reverse Primer 5′ > 3′	Template Size (Base Pairs)
E-cadherin	CAAATCCAACAAAGACAAAGAAGGC	ACACAGCGTGAGAGAAGAGAGT	1 ea @ 25 (FWD), 22 (REV) bases
N-cadherin	CATCATCATCCTGCTTATCCTTGT	GGTCTTCTTCTCCTCCACCTTCT	1 ea @ 24 (FWD), 23 (REV) bases
TWIST1	ATGGCAAGCTGCAGCTATG	AGTTATCCAGCTCCAGAGTC	1 ea @ 19 (FWD), 20 (REV) bases
SLUG	AGCATTTCAACGCCTCCAA	ACACAGTGATGGGGCTGTAT	1 ea @ 19 (FWD), 20 (REV) bases
SNAIL1	GAAAGGCCTTCAACTGCAAA	TGACATCTGAGTGGGTCTGG	1 ea @ 20 (FWD), 20 (REV) bases
SOX2	CATCACCCACAGCAAATGAC	CAAAGCTCCTACCGTACCACT	1 ea @ 20 (FWD), 21 (REV) bases

**Figure A7:**
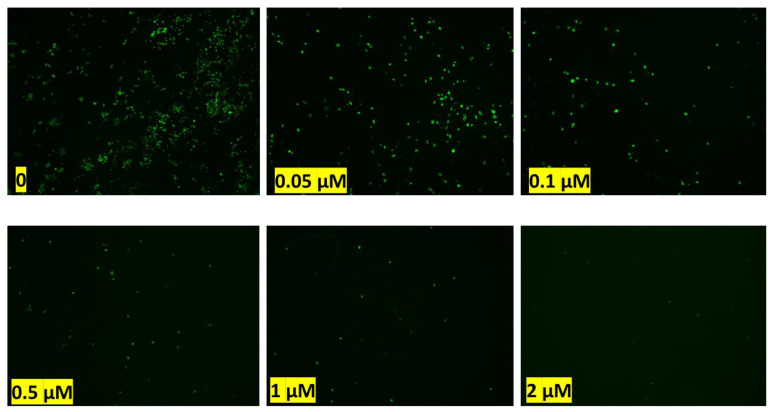
Effect of Doxorubicin on tumor cell (MDA-MB 231) migration after 5 days: green dots are the tumor cells moved out of the hydrogel and migrated to the top surface of scaffolds after 5 days of treatment with different doses of Doxorubicin.

**Figure A8:**
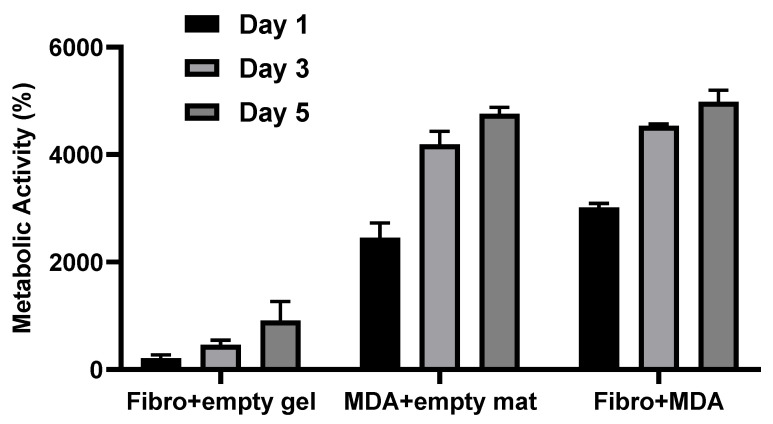
Cellular metabolic activity in different environments including fibroblast stromal cells alone on the mat with empty gel, MDA-Mb 231 tumor cells embedded in gel overlaid an empty mat, and co-culture of fibroblast and MDA-MB 231 in the 3D model (combination of mat and gel).

## Data Availability

The data presented in this study are available on request from the corresponding author.

## Appendix A

Here is a section as supplementary data added to present relevant results regarding the general aspects of the current innovative idea.

- Stromal cell distribution on and inside the mats:
  1. By florescent microscopy on the top surface of the mats:

2. By confocal imaging through the depth of mats at day 0 (penetration depth: max 60 μm after 30 min incubation with cells) and at day 7 (penetration depth: max 120 μm):

- Confocal images showing tumor cell distribution inside A1G7 hydrogel over 5 days:

- Evaluation of cell viability on plasma-treated mesh and inside hydrogel

As indicated below in Figure A5, viability of cells after 7 days was over 95% both in hydrogels and on the mesh.

- Doxorubicin screening tests in PP-3D-S versus 2D model:

- Imaging tumor cells migrated onto the mats with and without Doxorubicin treatment:

- Actual metabolic activity of tumor cells compared with stromal cells cultured in PP-3D-S model over the course of 5 days:
